# InpactorDB: A Classified Lineage-Level Plant LTR Retrotransposon Reference Library for Free-Alignment Methods Based on Machine Learning

**DOI:** 10.3390/genes12020190

**Published:** 2021-01-28

**Authors:** Simon Orozco-Arias, Paula A. Jaimes, Mariana S. Candamil, Cristian Felipe Jiménez-Varón, Reinel Tabares-Soto, Gustavo Isaza, Romain Guyot

**Affiliations:** 1 Department of Computer Science, Universidad Autónoma de Manizales, 170002 Manizales, Colombia; paula.jaimesb@autonoma.edu.co (P.A.J.); mariana.candamilc@autonoma.edu.co (M.S.C.); 2 Department of Systems and Informatics, Universidad de Caldas, 170002 Manizales, Colombia; gustavo.isaza@ucaldas.edu.co; 3 Department of Physics and Mathematics, Universidad Autónoma de Manizales, 170002 Manizales, Colombia; cristian.jimenezv@autonoma.edu.co; 4 Department of Electronics and Automation, Universidad Autónoma de Manizales, 170002 Manizales, Colombia; rtabares@autonoma.edu.co; 5 Institut de Recherche pour le Développement, CIRAD, University of Montpellier, 34394 Montpellier, France

**Keywords:** LTR retrotransposons, machine learning, deep neural networks, bioinformatics, plant genomes, genomics, InpactorDB

## Abstract

Long terminal repeat (LTR) retrotransposons are mobile elements that constitute the major fraction of most plant genomes. The identification and annotation of these elements via bioinformatics approaches represent a major challenge in the era of massive plant genome sequencing. In addition to their involvement in genome size variation, LTR retrotransposons are also associated with the function and structure of different chromosomal regions and can alter the function of coding regions, among others. Several sequence databases of plant LTR retrotransposons are available for public access, such as PGSB and RepetDB, or restricted access such as Repbase. Although these databases are useful to identify LTR-RTs in new genomes by similarity, the elements of these databases are not fully classified to the lineage (also called family) level. Here, we present InpactorDB, a semi-curated dataset composed of 130,439 elements from 195 plant genomes (belonging to 108 plant species) classified to the lineage level. This dataset has been used to train two deep neural networks (i.e., one fully connected and one convolutional) for the rapid classification of these elements. In lineage-level classification approaches, we obtain up to 98% performance, indicated by the F1-score, precision and recall scores.

## 1. Introduction

Transposable elements (TEs) have key roles in plant genomes. They are major contributors to genomic size [1,2], rearrangement events (such as fissions, fusions, and translocations) [3], chromosome organization and structure (e.g., centromeres) [4], and evolution and adaptation to the environment [5]. These dynamic elements can be activated under several biotic or abiotic stresses, such as pathogens [6,7], defense-associated stresses [8], heat, drought and salt stresses, freezing, polyploidization and hybridization events [9,10], UV light [11], and X-ray irradiation [12]. Transposable elements are also known to participate in reproductive isolation between genotype of the same species (reviewed in [13]) [14] and to shape the genome architecture during the process of plant speciation [15].

TE classification is still a subject of debate, despite the fact that a standard has emerged. TE classification is generally performed hierarchically [16], whereby TEs are first divided into classes according to their replication cycle: Class I or retrotransposons, which follow a copy-and-paste strategy using an RNA intermediate; and Class II or DNA transposons that use a cut-and-paste mobility mechanism through a DNA molecule [17]. Next, TE levels correspond to orders, superfamilies, lineages (also called families), and sub-families [18]. Among these, long terminal repeat (LTR) retrotransposons (LTR-RTs, an order of retrotransposons) are the most abundant TEs in plant genomes [19,20] and can account for up to 80% of the plant genome size, such as in wheat, barley, or rubber tree [21]. LTR-RTs are characterized by the presence of one or several open reading frames involved in the mobility of the element, flanked by a direct tandem repeat of 100 pb to more than 5000 bp, called LTR. These LTRs are directly involved in the transcription regulation of the element by the host’s machinery [22,23].

LTR-RT in plants are classically divided into two major superfamilies: Copia (also called Ty1) and Gypsy (also called Ty3), based on the organization of coding domains in the element [24,25]. Each superfamily is sub-classified into lineages or families according to coding region similarities and phylogenetic relationships of the reverse transcriptase (RT) domains, a combination of several domains or the complete polyprotein of the elements [24,26,27]. Llorens and coworkers [28,29] classified LTR-retrotransposons based on a phylogenetic analysis of 268 non-redundant element and in plants, 5 Copia and 2 Gypsy lineages have been identified, and further sub-classified into clades. With a bigger sampling composed of 5410 Copia and 8453 Gypsy elements from 80 plant genomes and a phylogenetic approach, Neumann and coworkers identified 16 Copia (that is, Ale, Alesia, Angela, Bianca, Bryco, Lyco, Gymco-I,II,III, IV, Ikeros, Ivana, Osser, SIRE, TAR and Tork), and 14 Gypsy lineages, sub-divided into chromovirus and non chromovirus elements (that is, CRM, Chlamyvir, Galadriel, Tcn1, Reina, Tekay, Athila, Tat-I,II,III, Ogre, Retand, Phygy and Selgy) [30]. Coding domains of these classified elements are available as curated libraries (Gypsydb and RexDB) for fine annotation of elements using homology based software such as RepeatMasker [31].

The classification of LTR-retrotransposons as deep as the classification in lineages finds its justification in numerous studies showing the dynamics of amplification of these elements. For example, in some plant genomes, sudden expansion of genome size is the result of the amplification of one or a small number of lineages [32,33,34]. Different copy number, amplification history and chromosomal distribution of lineages shape the genome architecture of plants [35,36,37]. A better fine-scale annotation of LTR retrotransposon in plants will likely reveal new lineage-specific mechanisms of genome size variation and divergence. Currently, a challenge in genomics is to reliably annotate TEs. These elements have certain characteristics that make their identification and classification a complex task [38,39], such as repetitiveness, structural and nucleotide diversity, complex mobilization dynamics (including nested insertions), and species specificity [18,40,41]. Although de novo, homology-based, structure-based, and comparative genomics bioinformatics methods (or a combination of several methods) can automatically detect and classify TEs [42] (for a review see [5,26]), all of these approaches have limitations due to the diversity of TE structures, the quality of genome assemblies into others, and the sole use of any of these cannot produce high quality results. Thus, the TE annotation process usually relies on much manual work done by experts [43]. With the recent advances of sequencing technologies, many plant genomes have been sequenced and the automation of TE annotation is needed to process the large amount of DNA sequence data [44]. Recent studies have demonstrated that machine learning (ML) can be applied to automatically annotate or even to both identify and annotate TEs in short times [18,45,46,47,48] using publicly available databases such as Repbase [49], PGSB [50], RepetDB [44], among others (for a list see [5]). Despite the available datasets, none of these attains a lineage-level classification and several do not include plant species from certain families, which could affect the generalization performance of the ML-based algorithms.

In this work, we present InpactorDB, a semi-curated dataset comprising more than 130,000 LTR retrotransposons from 195 plant species belonging to 108 families. These elements are classified to the lineage level and are filtered by length, number of coding domains present, nested insertion of class II TEs, and other retrotransposons. In addition, we removed the redundancy of elements through consensus creation following the same methodology of REPET, obtaining more than 67,000 sequences. This dataset constitutes a valuable resource for homology-based TE annotation, which is the most used approach [39], such as in RepeatMasker. Furthermore, this database also contributes to developing and testing ML-based algorithms for alignment-free and automatic annotation methods. Finally, we tested InpactorDB using two currently available deep neural networks for the classification of LTR retrotransposons to the lineage level.

## 2. Materials and Methods

### 2.1. Databases and LTR-RT Classification Processes

We collected information about LTR retrotransposons from three known TE databases: Repbase (v. 20.05, 2017) [49], PGSB [51], and RepetDB [44]. In addition, we detected LTR retrotransposons using different tools that follow a structure-based identification strategy. First, we used LTR_STRUC [52], due to its low level of false positive rates in plants (Romain Guyot, personal communication), on 69 available plant genomes to produce a dataset named here as “LTR_STRUC”; however, LTR_STRUC can be run only under Windows XP and takes a considerable execution time. Therefore, we used EDTA v1.9.3 [53], which uses LTR_Finder [54], LTRharvest [55], and LTR_retriever [56], to detect LTRs in 87 additional species (Supplementary Material S1) and generated a new dataset called “EDTA”. For Repbase, we joined the LTR domains and the internal section (concatenating before and after) of each LTR retrotransposon into a single sequence. Plant genomes to be analyzed were selected to target 103 different Angiosperm species families and in priority assemblies with low genome size (Supplementary Material S1).

**Figure 1:**
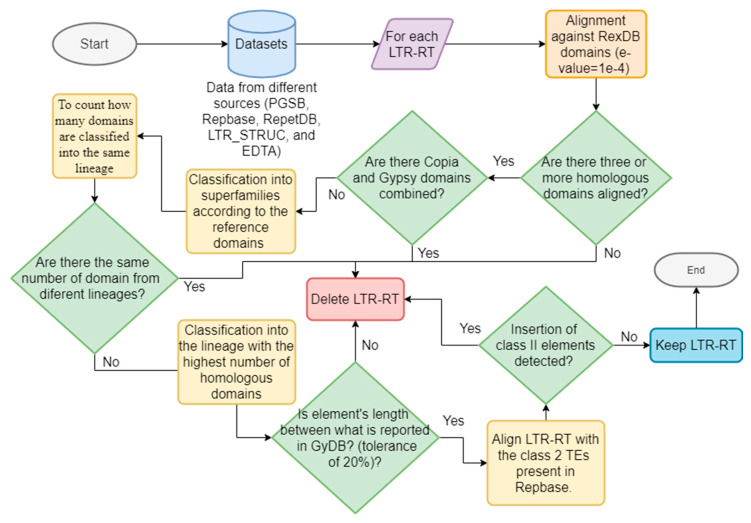
Schematic representation of the classification and filtering process performed for InpactorDB.

We applied the methodology proposed by Inpactor [57] to classify the elements of all all the datasets. Inpactor uses a homology-based strategy with known coding domains belonging to LTR-RTs; specifically, we used the RexDB [27] domain library as the reference. LTR-RTs were classified into superfamilies [for example, Gypsy (RLG) or Copia (RLC)] and sub-classified into lineages according to the similarities of five amino acid reference domains (GAG, AP, RT, RNAseH, and INT domains [58]). In addition, we applied filters to keep only intact elements by removing (1) predicted elements with domains from two superfamilies (that is, Gypsy and Copia, potential chimeric elements), (2) elements with domains belonging to two or more lineages, (3) elements with lengths different than those reported by the Gypsy Database (GyDB) [29], with a tolerance of 20%, (4) incomplete elements with less than three identified domains, and (5) elements with insertions of class II TEs (reported in Repbase). Figure 1 shows a general representation of the classification and filtering process.

The data generated is available at Zenodo under doi:10.5281/zenodo.4453481 and at DataSuds (https://dataverse.ird.fr) under doi:10.23708/QCMOUA.

### 2.2. Statistical Analysis

The datasets used in this study have different origins and characteristics such as the type of sequences (that is, consensus or individual DNA sequences) and pre-processing (that is, curated or non-curated sequences). We used ML algorithms such as logistic regression (LR), linear discriminant analysis (LDA), K-nearest neighbors (KNN), multi-layer perceptron with one layer (MLP), random forest (RF), decision trees (DT), naïve Bayes network (NB), and support vector machine (SVM) to test the performance of the datasets. We used the F1-score as the performance metric, which is the harmonic mean of precision and sensitivity [39] and we used it as the accuracy indicator; we used k-mer frequencies with 1 ≤ k ≤ 6 as features, and we used scaling and dimensional reduction using principal component analysis (PCA) as pre-processing steps, according to [39].

We created subsets of the datasets according to their characteristics (Table 1). To avoid bias related to the number of elements in each subset, we randomly selected the same number of LTR retrotransposons of each lineage that was present in the smallest dataset (Repbase; ~2842 elements).

A one-way analysis of variance (ANOVA), using subset type as the variable factor, was conducted to determine statistically significant differences between the performances of the algorithms applied to the subsets (Table 1). Additionally, we performed the Shapiro–Wilks normality test on the standardized residuals and a homoscedasticity test through the Bartlett test to determine the need for a non-parametric framework, such as the Kruskal–Wallis test.

Given significant statistical differences, a post hoc test was performed to identify which datasets generated these differences. This test was based on pairwise comparisons using Bonferroni’s method in the non-parametric framework of Duncan’s method. The pairwise comparisons were conducted as follows:

$$H0:\;\mu_{i}=\mu_{j}$$

(1) $$H1:\;\mu_{i}\neq \;\mu_{j}$$

Following the pairwise analysis, we selected a subset of the data that did not display statistically significant differences in the performance of ML algorithms with the other subsets, but taking into account that the average performances are the best among the other subsets.

### 2.3. Post-Processing and Generalization Tests through Deep Neural Networks

Based on the results of significant differences, we removed the redundancy of LTR retrotransposon sequences in the individual genomic datasets (PGSB, LTR_STRUC, and EDTA). For this, we used the same methodology implemented in REPET. First, we performed a BLASTN v2.4.0 (NCBI-Blast) [59] of all elements against all (separating each dataset) using an evalue = 1 × 10^−300^ and an identity cutoff ≥90. Then, we clustered the sequences using Silix v1.2.11 [60] with a minimum length of 95% and a minimum identity of 90%. Next, we generated a multiple alignment of each group using MAFFT v7.305b [61] and removed the columns in which all but one sequence showed gaps, using trimal v1.2 [62]. Finally, we built consensus sequences based on the majority system using cons (EMBOSS v6.5.7 [63]). This dataset is referred to as the non-redundant version of InpactorDB.

**Figure 2:**
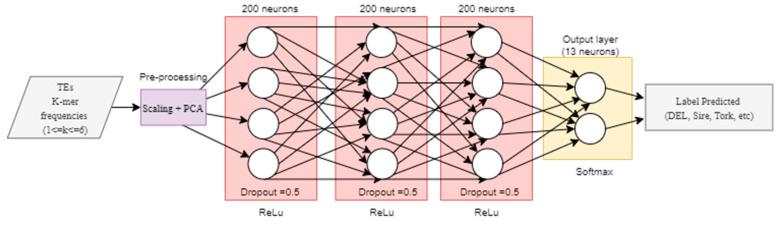
Implementation of the fully connected neural network architecture proposed by Nakano et al. [46].

We used the non-redundant version of InpactorDB to explore the automatic alignment-free classification process of LTR retrotransposons to the lineage level. Then, we implemented two deep neural networks (DNN) based on previously published research. First, we tested the hyper-parameter values proposed by Nakano et al. [46] for a fully connected DNN to classify TEs into superfamilies following a hierarchical strategy. The network had three hidden layers with 200 neurons each. The training stage was performed using 200 epochs (times that the entire training set is used to train the network) and mini batches of size 128, stochastic gradient descent (SGD) with a learning rate of 0.01, and Adam as the optimizer with a learning rate α = 0.001, β1 = 0.9, β2 = 0.999, and ε = 10^−8^, where β1 and β2 are the first and second exponential decay rate of the moment vector, respectively. The loss function used was the mean squared error and the activation function was ReLu (Figure 2).

**Figure 3:**
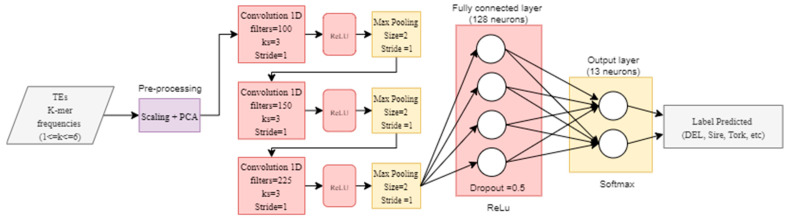
Implementation of the convolutional neural network (CNN) architecture proposed in DeepTE [48].

We also implemented a convolutional neural network (CNN) using the hyper-parameters proposed in DeepTE [48], which was previously used to classify TEs from all classes (that is, retrotransposons and DNA transposons) into superfamilies. This CNN consisted of three layers with 100, 150, and 225 filters with a kernel size of 3. Max pooling was used after each convolutional layer with a window size of 2. A dropout of 0.5 was used after the last convolutional layer. Finally, a fully connected layer with 128 units was used, and a softmax output layer was set to calculate the probabilities of the predicted classes. ReLu was used as the activation function in the three convolutional layers and the fully connected layer. Furthermore, we used a categorical_crossentropy loss function and an ADAM optimizer with a learning rate of 0.001 (Figure 3). The implementation of these DNN architectures in Python and Tensorflow 2 (Keras) can be consulted in Supplementary Material S2.

As features, we used k-mer frequencies with 1 ≤ k ≤ 6. We also applied scaling and reduction of dimensionality using principal components analysis (PCA) as suggested by [39]. Each dataset was partitioned into 80% for training, 10% for validation, and 10% for testing. We measured the generalization performance using LTR retrotransposons from the genomes of Gardenia jasminoides [64], Daucus carota [65], Abrus precatorius [66], and Asparagus officinalis [67], which were not included in the training dataset. These LTR retrotransposons were detected using EDTA and were processed with the same pipeline used for the elements in InpactorDB (Figure 1). These genomes were downloaded from NCBI (assemblies: ASM1310374v1, ASM162521v1, Abrus_2018, and Aspof.V1). We used all lineages, except those absent from Angiosperms, like Chlamyvir, Tcn1, Phygy, Selgy, TatI,II,III, Osser, Bryco, Lyco, GymcoI,II,III, and IV. In addition, considering their close relationships, we decided to merge Tar and Tork groups into the Tar/Tork group, Ivana and Oryco into the Ivana/Oryco group, and Ogre and Retand into TAT groups [27]. For a better visibility of the lineage names, we renamed Ale as Ale/Retrofit, Tekay as Del/Tekay and Ivana as Ivana/Oryco.

All the experiments were performed using Python 3.6, Scikit-Learn library 0.22 [68] for pre-processing, data partition, and ML algorithms, and tensorflow 2 (keras) [69] for deep neural networks, installed in an Anaconda environment in a Linux operating system over GPU. We ran our tests using the HPC clusters of the Institut Français de Bioinformatique (https://www.france-bioinformatique.fr), IRD (https://bioinfo.ird.fr/), and Genotoul Bioinformatics platform (http://bioinfo.genotoul.fr/), managed by Slurm, and in the BiRD platform (https://pf-bird.univ-nantes.fr/) and Migale Bioinformatics facility (http://migale.jouy.inra.fr/), managed by Sun Grid Engine (SGE).

## 3. Results

First, we downloaded 9278, 61,730 and 16,137 plant LTR-RT sequences from Repbase, PGSB, and RepetDB, respectively. Additionally, we identified 49,896 elements using LTR_STRUC and 221,052 elements using EDTA. However, EDTA did not predict full-length LTR-RT in eight plant genomes, namely *Calotropis procera*, *Spergula arvensis*, *Diospyros lotus*, *Magnolia ashei*, *Moringa oleifera*, *Passiflora edulis*, *Rafflesia leonardi*, and *Aristotelia chilensis*. It is likely due to low N50 of some assemblies (below 10kb for *S. arvensis*, *D. lotus*, *M. ashei*, *P. edulis*, *R. leonardi*, and *A. chilensis*) and/or the absence of full-length copies of LTR-RT.

**Figure 4:**
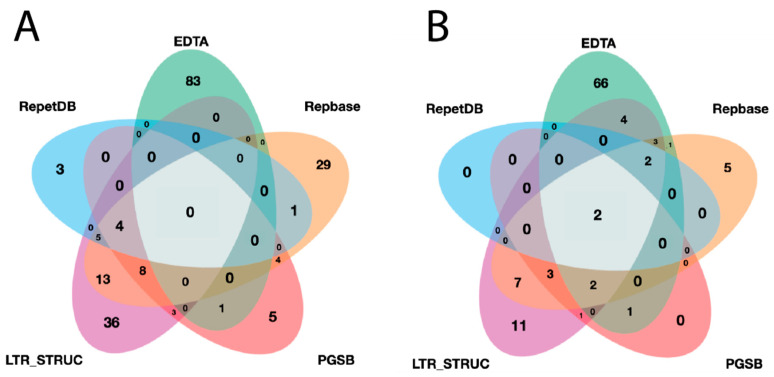
Venn diagrams representing: (**A**) the number of unique and shared plant species between datasets and (**B**) the number of unique and shared plant families between datasets.

A lineage-level classification process was performed for all identified elements and a filtration process was applied. The final redundant library of InpactorDB comprised 130,439 elements from 195 plant species belonging to 108 Angiosperm families (Figure 4, Supplementary Material S3).

### 3.1. Analysis of Significant Differences

In order to reduce the number of sequences in InpactorDB without losing representativeness and to increase data quality, we used datasets with different characteristics (that is, consensus versus individual genomic sequences and curated versus non-curated sequences) to train the ML algorithms. Using the sequences retained after filtering, we performed an analysis of significant differences to determine if the dataset characteristics (Table 1) affected the performance of eight ML algorithms (LR, LDA, KNN, MLP, RF, DT, NB, and SVC) using k-cross validation with k = 10 (Supplementary Material S4). We could not assume normality of the data or homogeneity of variances; therefore, a non-parametric Kruskal–Wallis test was conducted.

The Kruskal–Wallis test showed a *p*-value lower than 2.2 × 10^−16^. Due to the non-normal distribution of the data, a non-parametric pairwise comparison test was applied using Bonferroni’s method through a Duncan’s range test (Table S1). Table 2 shows the results from the subsets with the best performances. We found no differences between the curated, consensus, PGSB, and RepetDB subsets regarding the performance of the ML algorithms. Since the curation process is more complex than building consensus sequences, we concluded that it is better to remove redundancy through consensus.

### 3.2. Post-Processing and Classification Using Deep Neural Networks

The methodology used by REPET to build consensus sequences from TEs was applied to our full dataset. Since two datasets are already consensus sequences, we only applied this process to PGSB, LTR_STRUC, and EDTA datasets of InpactorDB. After consensus creation, we reduced the number of elements to 9608 (6X reduction), 22,530 (6X reduction), and 26,915 (8X reduction) for PGSB, LTR_STRUC, and EDTA subsets, respectively.

The final non-redundant version of InpactorDB consists of 67,241 LTR retrotransposons. Both redundant and non-redundant versions of InpactorDB are available in FASTA format, in which the sequence identifiers have the following general identification code:

>Superfamily-Lineage-plant_family-specie-source-length-ID,

where Superfamily is either RLC (for Copia) or RLG (for Gypsy), Lineage (or family) following the RexDB nomenclature, source (Repbase, RepetDB, PGSB, LTR_STRUC or EDTA datasets), length, and ID, a unique number that identifies each element in InpactorDB. All those fields are separated by a dash character and composed names (as in species) are separated by an underscore character.

The number of consensus sequences for each lineage is unbalanced, probably reflecting the diversity of subfamilies for several lineages (Table 3). Ivana, and Ikeros are the most frequently found lineages in InpactorDB, while TAT, Retrofit and DEL are lineages with the greatest number of elements.

Using the non-redundant version of InpactorDB, we tested two published deep neural networks, which were used to classified TEs from all orders into superfamilies. First, we used an FNN architecture that applied a hierarchical approach to classify TEs [46]. We also tested a CNN published by [48]. We implemented these architectures using Python 3, Tensorflow 2, and Keras (Supplementary Material S2) with hyper-parameters published by their authors. Both architectures require only 25 epochs to achieve high performance.

**Figure 5:**
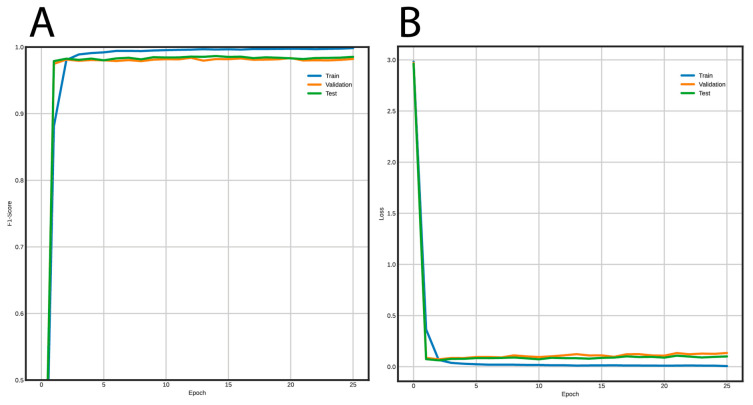
Learning curves using the FNN architecture to classify long terminal repeat (LTR) retrotransposons into lineages. (**A**) F1-Score vs. epochs, and (**B**) loss vs epochs.

**Figure 6:**
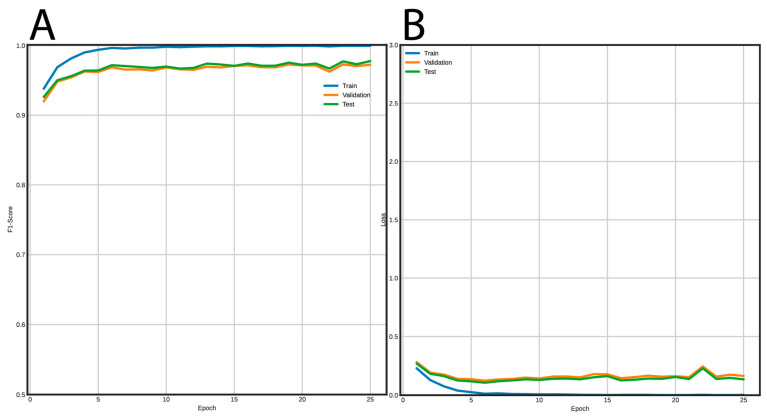
Learning curves using the CNN architecture to classify LTR retrotransposons into lineages. (**A**) F1-Score vs epochs, and (**B**) loss vs epochs.

Figure 5 shows the training curves for the FNN and Figure 6 for the CNN. Using the FNN, we obtained 98% accuracy, F1-Score, recall, and precision with the validation and test datasets. On the other hand, the CNN had a performance of 97% for the same metrics using the validation and test datasets.

Most lineages were correctly classified by both DNN architectures, which achieved performances of up to 99–100% (Table 4 and Table 5). The lowest F1-Score was found for the Ikeros lineage, likely given the low number of sequences of this lineage in InpactorDB (7).

To test the accuracy of the FNN and CNN under more realistic conditions of LTR-RT classification, we downloaded four plant genomes from species and genera that were not present in our dataset. We selected Gardenia jasminoides, a plant from the Rubiaceae family (Asterids), Daucus carota from Asterids, Abrus precatorius from Rosids, and Asparagus officinalis from monocots. EDTA detected 2648, 1167, 851, and 4692 intact LTR retrotransposons, respectively, and 1010, 628, 579, and 2677 were kept after applying filters, respectively. Using the parameters learned using InpactorDB, the FNN and CNN displayed F1-scores of 97.8% and 86.5% for Gardenia jasminoides, 98.8% and 95.5% for Daucus carota, 99% and 98.1% for Abrus precatorius, and 93.4% and 86.4% for Asparagus officinalis, respectively.

## 4. Discussion

Given the increasing amount of sequencing data in plants, there is a need to find an automated and rapid way to annotate transposable elements that make up the main part of their genomes. More particularly, LTR retrotransposons constitute the majority of plant DNA (up to 85%) [70] and have crucial roles in genome evolution size, dynamics [71,72] and chromosome organization [3,73]. Furthermore, the high quality detection of TEs improves the accuracy of coding region annotations and functional gene studies [5,74]. Moreover, each lineage of LTR retrotransposons has different dynamics and chromosomal distribution [24,75], and represents different fractions of the genome [5]. For instance, Copia elements are more frequently observed in euchromatin [73,76] and Gypsy retrotransposons are mainly found nested in heterochromatin regions [77,78]. Thus, the classification of TEs, especially, LTR-RTs, into superfamilies and lineages is crucial to better understanding genome dynamics.

Current computational tools apply several strategies to detect and classify TEs, which can be grouped into homology-based, structure-based, de novo, and those based on comparative genomics [42,46,79,80]. Nevertheless, all these strategies have limitations, such as the dependance on high quality species-specific TE libraries for a homology-based strategy, the incorporation of host multigene families as repeats [26], and the low quality identification for partial or degenerated elements with a structure-based strategy, as well as others [5]. Indeed, the quality of the genome assembly can deeply influence the quality of the detection and the classification.

For detecting LTR-RTs, current tools commonly use structure-based searches in order to take advantage of the well-established features of these kinds of elements. For deep annotation and classification (specifically, classification into linages/families), the homology-based approaches are actually the most frequently used [46]. Since homology-based methods detect TEs based on their similarity to reference TE sequences [81], the quality of the entire process depends on the utilization of a well curated and extensive library or database of TEs. Different plant TEs databases are available (for a list, see [5]), which contain consensus [44,49,82] or genomic [51,83] TE sequences and peptides of coding domains [27,84].

Although there are several databases comprising thousands of TEs, the great structural diversity of these repetitive elements and their species-specificity requires a library with reference LTR retrotransposons from a high number of species from different plant families. For example, PGSB contains 50,000 LTR-RTs from ~60 plant families, RepetDB has ~16,000 from 13 plant species, and Repbase contains ~9700 LTR-RTs from ~70 plant species.

Unlike Repbase, PGSB, and RepetDB, InpactorDB only contains intact full-length LTR-RTs. All elements in this dataset have passed several filters to keep as much as possible LTR retrotransposons that can be used as references. We removed sequences shorter or larger than lengths published by the Gypsy Database (with a tolerance of 20%) to discard incomplete sequences (due to internal deletion for example) and LTR-RTs with nested insertions. Then, we deleted elements with combined domains reported in LTR-RTs from different superfamilies (Copia or Gypsy), suggesting chimeric elements. We also removed ambiguous classified elements with the same number of domains from two or more different lineages (for example, those with two domains from DEL and two domains from REINA lineages). These filters were designed to keep as much as possible LTR-RTs with no nested insertions by other LTR retrotransposons. Finally, we discarded elements with insertions of class II TEs (present in Repbase) to retain putative intact sequences of LTR-RTs.

The currently available databases are valuable resources to annotate LTR-RTs in plant genomes; however, they constitute a small fraction of all sequenced plant species that are representative of plant families. In the data-driven science era, the creation and release of datasets are considered crucial tasks due to their importance in the performance of ML algorithms. A dataset containing repetitive elements from a high diversity of plant species and families is required for tasks regarding LTR retrotransposons, given their natural properties. Thus, more extensive datasets, for training a ML model, could improve its performance and, especially, its generalization because using a data set with more samples for each class (family/lineage) gives the algorithm more information about how the sequences of the LTR-RTs are regardless of the species they come from, reducing the probability of overfitting the model to a certain set of species. Therefore, the algorithm will be more likely to make accurate predictions in genomes that it has never seen before. In an automatic-annotation tool of LTR-RTs, generalization is required since it will be trained using currently sequenced genomes, but it will be used to annotate elements from newly sequenced plant species, which were potentially absent in the training set. Consequently, datasets that include species from a higher number of plant families and genera could improve the probability of the ML algorithm to predict LTR-RTs from new species. Given this, our main aim was to create a dataset comprising LTR-RTs from different plant species and families; accordingly, InpactorDB contains LTR retrotransposons from 25, 13, and 64 plant species from PGSB, RepetDB, and Repbase, respectively, with additional elements from 69 and 84 plant species using LTR_STRUC and EDTA, respectively. By joining all of these libraries, our dataset consists of more than 130,000 LTR retrotransposons in the redundant version. We observed that consensus and curated databases have the best performance on average for training ML algorithms, with no significant differences between the two. Thus, we applied the process for consensus creation implemented by REPET to build a non-redundant version of InpactorDB with 67,241 elements.

Currently, there is no definitive consensus regarding the LTR-RT classification and naming systems for RT-LTRs. Although many studies use the hierarchical classification of Wicker et al. [16] there is still debate and disagreement on the taxonomy and naming of LTR-RT at different levels of classification [27,29]. Common initiatives are needed for a single classification and naming system at the international level. Our study is based on a robust phylogenetic approach using protein domains for classification [27]. However, as that study used only 56 different plant genomes for the phylogeny of LTR-RT, it cannot be excluded that the diversity of LTR-RT is more complex in plants. An incomplete classification could probably impact automatic classification approaches. Recently, Neumann et al. [27] proposed the separation of several lineages of LTR-RTs based on phylogenetic analyses done with 80 plant genomes. Some of the new lineages appear unevenly distributed among plants families. Clamyvir and Osser are specific of Chlorophyta (a taxon of green algae), while Phygy and Bryco, are specific to Bryophyta (non-vascular land plants), Selgy TatI, Lyco are specific to Lycopodiophyta and finally Gymco is specific to Acrogymnospermae. These lineages are not present in Angiosperm species and as a consequence were not studied here. Interestingly, other lineages show a low number of predicted copies in angiosperm species like Ikeros (84), in the non-redundant version of InpactorDB (Table 3).

Ivana (68) demonstrated very few copy numbers in plant species in the non-redundant version of InpactorDB. In contrast, ALE/Retrofit, TAT, and DEL/Tekay accounted for a large number of samples in the dataset with 12,026, 17,923, and 10,383, respectively. This large unbalance is inadequate for ML algorithms since the model will learn how to classify LTR-RTs from the most frequent lineages, but the performance will reduce in less frequent lineages (Table 4 and Table 5). Lineages present in non-angiosperm species were not considered in our DNN tests due to the few number of genomes available in the databases (69 until 2019, where 55 corresponded to green algae [85]), compared with 323 angiosperm genomes found in the databases until 2019 [85]. In the future, it will be essential to include more lineages from non-angiosperms when more genomes will be available for exhaustive classification in plants.

In the current (post) genomic era [86,87], there is a need for automating TE annotation [39,44] to quickly analyze the huge amount of genomic data. Machine learning algorithms have become popular in bioinformatics because they provide promising results in complex tasks and given the availability of large databases. InpactorDB is designed to be a useful tool in the detection and annotation of LTR retrotransposons using both homology-based software (such as RepeatMasker, Supplementary Material S5) and novel free-alignment algorithms based on ML. Using InpactorDB to train two DNN architectures, we obtained up to 98% F1-Score, precision and recall in the problem of classifying LTR retrotransposons into lineages. Additionally, we highlight that only 25 epochs were needed to achieve a good training performance and hyper-parameter tuning was not required. Using more than 4000 LTR-RTs from four plant species that were not included in InpactorDB, we achieved up to a 99% F1-Score using the FNN model, demonstrating good generalization performance. As future work, we propose the use of InpactorDB to generate a new DNN architecture that can improve the performance of the classification of LTR retrotransposons in plant genomes.

## 5. Conclusions

InpactorDB is a semi-curated dataset of LTR retrotransposons from 195 plant species representing 108 plant families. It comprises more than 130,000 (redundant) and over 60,000 (non-redundant) elements that are classified to the lineage level. InpactorDB was designed to be a tool to annotate LTR-RTs in plant genomes using homology-based algorithms, such as RepeatMasker, and to support automatic, ML-based, and alignment-free software, which is needed to process the large amount of genomic data produced by massive sequencing projects in the current post-genomic era. Given the high diversity of plant species and families contained in the dataset and the filters applied to the LTR-RT sequences, InpactorDB can be used as a basis to train ML algorithms or DNN architectures towards the implementation of automatic TE annotators in plant genomes.

## Figures and Tables

**Table 1:** Datasets used in the statistical analysis of machine learning (ML) performances.

Name	Observations
Repbase	Curated consensus sequences.
PGSB	Curated individual genomic sequences.
RepetDB	Non-curated consensus sequences.
LTR_STRUC	Non-curated individual genomic sequences.
Consensus	Union between Repbase and RepetDB.
Genomics	Union between PGSB and LTR_STRUC.
Curated	Union between Repbase and PGSB.
Non-curated	Union between RepetDB and LTR_STRUC.
All	Union between Repbase, PGSB, RepetDB, and LTR_STRUC.

**Table 2:** *p*-values obtained using pairwise comparison through Bonferroni’s method.

	Curated	Consensus	PGSB	RepetDB
Curated				
Consensus	1			
PGSB	1	0.188		
RepetDB	1	0.162	1	

**Table 3:** Number of elements for each lineage in the non-redundant version of InpactorDB.

Superfamilies	Lineages	Number of Sequences (Redundant)	Number Sequences (Non-Redundant)
Copia	ALE/RETROFIT	19,888	12,026
Copia	ANGELA	6889	1458
Copia	BIANCA	2872	1827
Copia	IKEROS	149	84
Copia	IVANA	88	68
Copia	ORYCO	6135	3468
Copia	SIRE	10,892	3130
Copia	TORK/TAR	11,460	6161
	**Total Copia**	**58,373**	**28,222**
Gypsy	ATHILA	6611	3499
Gypsy	CRM	4811	2134
Gypsy	DEL/TEKAY	18,330	10,383
Gypsy	GALADRIEL	1715	549
Gypsy	REINA	6387	4531
Gypsy	TAT	34,212	17,923
	**Total Gypsy**	**72,066**	**39,019**

**Table 4:** Performance obtained for each lineage using the FNN architecture.

Superfamilies	Lineages/Families	Precision	Recall	F1-Score	Support
Copia	ALE/RETROFIT	0.99	0.99	0.99	1220
Copia	ANGELA	0.96	0.98	0.97	145
Copia	BIANCA	0.99	0.99	0.99	166
Copia	IKEROS	0.67	0.57	0.62	7
Copia	IVANA/ORYCO	0.95	0.97	0.96	319
Copia	TORK/TAR	0.98	0.95	0.96	575
Copia	SIRE	0.99	0.98	0.99	325
Gypsy	CRM	0.98	0.97	0.97	201
Gypsy	GALADRIEL	1.00	0.93	0.96	58
Gypsy	REINA	0.99	1.00	0.99	497
Gypsy	TEKAY/DEL	0.99	0.99	0.99	1059
Gypsy	ATHILA	0.97	0.98	0.97	372
Gypsy	TAT	0.99	0.99	0.99	1787

**Table 5:** Performance obtained for each lineage using the CNN architecture.

Superfamilies	Lineages/Families	Precision	Recall	F1-Score	Support
Copia	ALE/RETROFIT	0.97	0.99	0.98	1220
Copia	ANGELA	0.96	0.94	0.95	145
Copia	BIANCA	1.00	0.95	0.98	166
Copia	IKEROS	1.00	0.43	0.60	7
Copia	IVANA/ORYCO	0.96	0.93	0.95	319
Copia	TORK/TAR	0.94	0.94	0.94	575
Copia	SIRE	0.99	0.97	0.98	325
Gypsy	CRM	0.97	0.92	0.94	201
Gypsy	GALADRIEL	1.00	0.74	0.85	58
Gypsy	REINA	0.98	0.99	0.98	497
Gypsy	TEKAY/DEL	0.98	0.98	0.98	1059
Gypsy	ATHILA	0.97	0.99	0.98	372
Gypsy	TAT	0.99	1.00	0.99	1787

## Data Availability

InpactorDB is available at Zenodo under the doi:10.5281/zenodo.4386317 and at Datasud (https://dataverse.ird.fr) under the doi:10.23708/QCMOUA.

## Supplementary Materials

The following are available online at https://www.mdpi.com/2073-4425/12/2/190/s1, Figure S1. Boxplot of F1-Score performance of all algorithms used different subsets, Table S1. Values obtained by pairwise comparisons using Bonferroni’s method. The colored cells have a *p*-value < 0.05, Supplementary Material S1: Assemblies used to find LTR-RTs with LTR_STRUC and EDTA, Supplementary Material S2: Jupyter Notebook of the implementation of both deep neural networks, Supplementary Material S3: Plant species and families of each dataset. Supplementary Material S4: F1-Scores of ML algorithms trained with each subset used in the statistical analysis. Supplementary Material S5: Repeat Masker annotation results of *Coffea canephora*, *Ananas comosus*, and *Oryza sativa* using as custom library InpactorDB, Repbase, and RepetDB.
