# Supplementary material for: InpactorDB: A Classified Lineage-Level Plant LTR Retrotransposon Reference Library for Free-Alignment Methods Based on Machine Learning
Source: Genes (Basel). 2021 Jan 28;12(2):190. doi: 10.3390/genes12020190 (PMC7910972; doi:10.3390/genes12020190)
Supplement: Supplementary file 1 [file genes-12-00190-s001.zip › Supplemental figures and tables.docx]

|  | From | To | Z | P.unadj | P.adj |
| --- | --- | --- | --- | --- | --- |
| 1 | All | Consensus | -4.1811 | 0.0000 | 0.0010 |
| 2 | All | Curated | -5.6597 | 0.0000 | 0.0000 |
| 3 | Consensus | Curated | -1.4786 | 0.1393 | 1.0000 |
| 4 | All | Genomic | -0.8442 | 0.3986 | 1.0000 |
| 5 | Consensus | Genomic | 3.3369 | 0.0008 | 0.0305 |
| 6 | Curated | Genomic | 4.8155 | 0.0000 | 0.0001 |
| 7 | All | LTR_STRUC | -0.1764 | 0.8600 | 1.0000 |
| 8 | Consensus | LTR_STRUC | 4.0047 | 0.0001 | 0.0022 |
| 9 | Curated | LTR_STRUC | 5.4833 | 0.0000 | 0.0000 |
| 10 | Genomic | LTR_STRUC | 0.6678 | 0.5042 | 1.0000 |
| 11 | All | non-Curated | -0.7051 | 0.4808 | 1.0000 |
| 12 | Consensus | non-Curated | 3.4760 | 0.0005 | 0.0183 |
| 13 | Curated | non-Curated | 4.9546 | 0.0000 | 0.0000 |
| 14 | Genomic | non-Curated | 0.1391 | 0.8894 | 1.0000 |
| 15 | LTR_STRUC | non-Curated | -0.5287 | 0.5970 | 1.0000 |
| 16 | All | PGSB | -6.9729 | 0.0000 | 0.0000 |
| 17 | Consensus | PGSB | -2.7918 | 0.0052 | 0.1887 |
| 18 | Curated | PGSB | -1.3132 | 0.1891 | 1.0000 |
| 19 | Genomic | PGSB | -6.1287 | 0.0000 | 0.0000 |
| 20 | LTR_STRUC | PGSB | -6.7966 | 0.0000 | 0.0000 |
| 21 | non-Curated | PGSB | -6.2678 | 0.0000 | 0.0000 |
| 22 | All | Repbase | -1.6325 | 0.1026 | 1.0000 |
| 23 | Consensus | Repbase | 2.5486 | 0.0108 | 0.3894 |
| 24 | Curated | Repbase | 4.0271 | 0.0001 | 0.0020 |
| 25 | Genomic | Repbase | -0.7883 | 0.4305 | 1.0000 |
| 26 | LTR_STRUC | Repbase | -1.4562 | 0.1453 | 1.0000 |
| 27 | non-Curated | Repbase | -0.9274 | 0.3537 | 1.0000 |
| 28 | PGSB | Repbase | 5.3404 | 0.0000 | 0.0000 |
| 29 | All | RepetDB | -7.0208 | 0.0000 | 0.0000 |
| 30 | Consensus | RepetDB | -2.8397 | 0.0045 | 0.1626 |
| 31 | Curated | RepetDB | -1.3611 | 0.1735 | 1.0000 |
| 32 | Genomic | RepetDB | -6.1766 | 0.0000 | 0.0000 |
| 33 | LTR_STRUC | RepetDB | -6.8444 | 0.0000 | 0.0000 |
| 34 | non-Curated | RepetDB | -6.3157 | 0.0000 | 0.0000 |
| 35 | PGSB | RepetDB | -0.0479 | 0.9618 | 1.0000 |
| 36 | Repbase | RepetDB | -5.3883 | 0.0000 | 0.0000 |

Table S1. Values obtained by pairwise comparisons using Bonferroni's method. The colored cells have a p-value < 0.05.


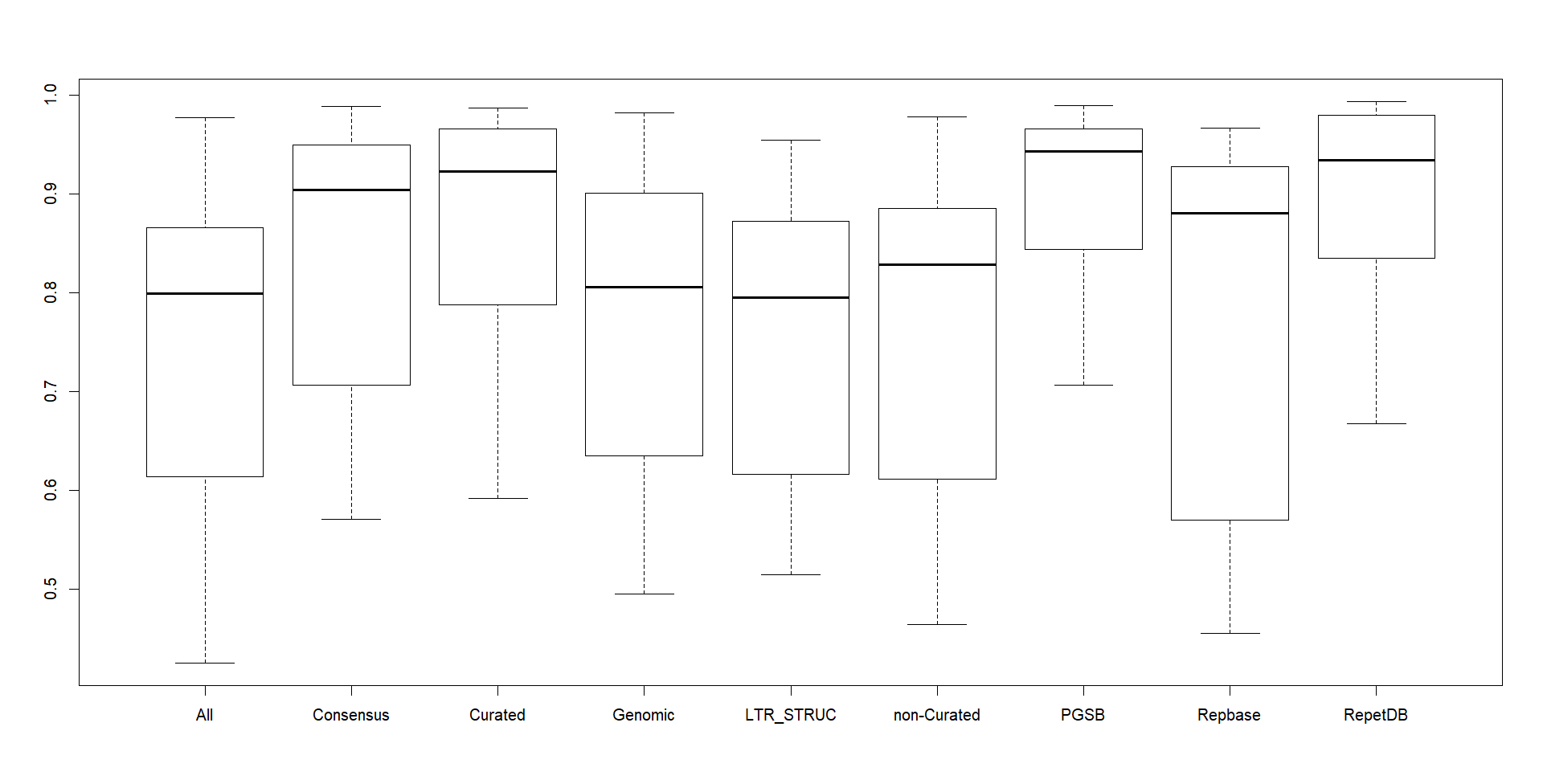


Figure S1. Boxplot of F1-Score performance of all algorithms used different subsets.
